# Incorporation of Ceragenins into Medical Adhesives and Adhesive Scar Tape to Prevent Microbial Colonization Common in Healthcare-Associated Infections

**DOI:** 10.3390/antibiotics13111002

**Published:** 2024-10-24

**Authors:** Aaron Zaugg, Elliot Sherren, Rebekah Yi, Alex Farnsworth, Fetutasi Pauga, Anna Linder, Lauren Kelly, Meg Takara, McKenna Hoather, Sierra Stump, Christine Behunin, Boston Boyack, Morgan Tolley, Kayla Holland, Morgann Salmon, Shenglou Deng, James E. Patterson, Paul B. Savage

**Affiliations:** 1 Department of Chemistry and Biochemistry, Brigham Young University, Provo, UT 84602, USA; aaronlz@student.byu.edu (A.Z.); ajfarnz@gmail.com (A.F.); mjsalmon@byu.edu (M.S.); james.patterson@byu.edu (J.E.P.); 2 Department of Molecular Pharmaceutics, University of Utah, Salt Lake City, UT 84112, USA

**Keywords:** ceragenins, polyacrylate adhesives, scar tape, healthcare-associated infections, methicillin-resistant *Staphylococcus aureus*, *Pseudomonas aeruginosa*, *Candida albicans*

## Abstract

**Background/Objectives:** Healthcare-associated infections involving surgical sites, skin trauma, and devices penetrating the skin are a frequent source of increased expense, hospitalization periods, and adverse outcomes. Medical adhesives are often employed to help protect compromised skin from infection and to secure medical devices, but adhesives can become contaminated by pathogens, exposing wounds, surgical sites, and medical devices to colonization. We aimed to incorporate ceragenins, a class of antimicrobial agents, into silicone- and polyacrylate-based adhesives with the goal of reducing adhesive contamination and subsequent infections. **Methods:** Three adhesives were developed and evaluated for the release of ceragenins, antimicrobial efficacy, adhesive strength, and dermal irritation. **Results:** Elution profiles over two weeks showed a high initial release followed by steady, long-term release. Standard microbial challenges of the adhesives by methicillin-resistant *Staphylococcus aureus*, *Pseudomonas aeruginosa*, or *Candida albicans* demonstrated microbial reduction for 6 to 68 days. Lap shear adhesive strength was not reduced for polyacrylate adhesives containing ceragenins, and no dermal irritation was observed in an in vivo model. **Conclusions:** Ceragenin-containing adhesive materials appear well suited for prevention of bacterial and fungal infections associated with medical devices and bandages.

## 1. Introduction

In 2022, the United States Center for Disease Control and Prevention (CDC) reported statistics from the National Healthcare Safety Network indicating that healthcare-associated infections (HAIs) such as central line-associated bloodstream infections and skin and soft tissue infections are responsible for 72,000 patient deaths annually in the United States [1,2,3]. Notably, surgical site infections (SSIs) are common and frequently lead to hospital readmission, with associated mortality in as many as 5% of cases [4]. Furthermore, the CDC considers many common HAI pathogens, including *Candida albicans*, *Pseudomonas aeruginosa*, and methicillin-resistant *Staphylococcus aureus* (MRSA), as urgent or serious threats. MRSA alone is globally responsible for 100,000 deaths annually, as well as almost USD 2 billion in healthcare costs according to a 2019 study [3,4].

Medical adhesives are ubiquitous in hospitals and healthcare facilities and are used to secure devices and lines extending through the skin, to protect compromised skin from microbial exposure, and/or to reduce surgical scarring [5,6,7]. However, these devices are also vectors for cross-contamination, and bacteria such as *P. aeruginosa* and *S. aureus* have frequently been cultured from medical adhesives. Fungal pathogens such as *Aspergillus flavus* and *C. albicans* have also been cultured from adhesive devices, including cardiac tape and cord catheters, with counts on covered skin being over one thousand times higher than those on control sites in some instances [5]. The problem is widespread, and some studies have found as much as 74% of catheter tape and 68% of operating room tape to be colonized before use [8,9].

The most commonly used polymers in pressure-sensitive adhesives are polyacrylates [10,11]. Polyacrylates can also be found in many different applications in the food industry, electronics, and environmental implementations [12]. Characteristics that make polyacrylates appealing for use in medical devices include their physicochemical abilities, such as elasticity, low cost, electrical conductivity, biocompatibility, and adhesion with moist tissue [12,13,14].

Silicone scar tape and other silicone-based adhesives are also routinely used in healthcare settings because they are able to repel moisture, are hypoallergenic, and have less peel force than traditional adhesives, reducing epidermal cell stripping and discomfort upon removal [15]. Silicone scar tape is also used to help with the aesthetics of repairing wounds, skin tears, blisters, allergic dermatitis, folliculitis, and moisture-associated skin damage [16]. One common use for scar tape is after cesarean deliveries, where the placement of either adhesive tape or tissue adhesive can help reduce the appearance of a potentially large scar [16]. From these incisions, scars can form when fibroblasts are unable to fully synthesize collagen and elastic fibers in the patient’s extracellular connective tissue [17]. Scar tape is used because it maintains hydration levels in the dermis, leading to the synthesis and release of collagen from fibroblasts [18].

In addition to natural flora, human skin is routinely exposed to diverse microbes. While allowing for a skin microbiome, microbial growth in human skin is controlled through innate immunity, with the primary active components comprising antimicrobial peptides (AMPs). These peptides are constitutively expressed by keratinocytes and are upregulated in damaged tissues [19]. When a medical device is placed near or in contact with the skin, it becomes a nidus for microbial growth because, in general, devices come without the innate immune function provided by human skin. Adhesives, whether polyacrylate- or silicone-based, can serve as such abiotic surfaces that pathogens can colonize; consequently, there is a need to protect these materials with innate immune-like antimicrobial properties. Adhesives have previously been used to deliver biologically active or medically relevant compounds with broad structural variety, and incorporating a stable and readily soluble antibiotic into an adhesive to control microbial colonization is a promising avenue for mitigating HAIs [20].

AMPs are attractive candidates for incorporation into adhesives for multiple reasons. They display activity against a range of bacteria, fungi, viruses, and parasites, making them optimal for healthcare settings where broad-spectrum protection is required [19]. Because they are present on skin, their use would be unlikely to introduce allergic reactions or to generate drug resistance. Additionally, AMPs have been associated with wound healing, and many are capable of recruiting cells and inducing proliferative, angiogenic, and immunomodulatory effects in wound sites [19]. AMPs also play roles in myofibroblast differentiation and the remodeling of the extracellular matrix, contributing to scarring [21]. However, AMPs are susceptible to proteolytic degradation and are costly to employ, resulting in a need for cheaper alternatives that play a similar protective role for medical devices.

Ceragenins are non-peptide mimics of AMPs and offer the advantages of AMPs without protease susceptibility and the relatively high costs of production. These molecules reproduce the amphipathic nature of AMPs through positively charged amines extending from a cholic acid backbone [22,23]. By mimicking the morphology of AMPs, ceragenins display broad-spectrum antimicrobial activity while posing minimal risk to the patient [24]. Ceragenins are also relatively cheap to produce and are stable at physiological temperatures, potentially allowing for economical production, longer storage, and broader applications in medical settings. The aim of this work was to evaluate the effectiveness of ceragenins incorporated into medical adhesives in preventing the growth of pathogens on and around adhesive materials.

## 2. Results

### 2.1. Development of Antimicrobial Adhesives

Ceragenins have previously been studied in medical applications, with ceragenins CSA-44 and CSA-131 being leading candidates for use as stand-alone antibiotics and for use in medical device coatings. CSA-131 is a representative ether-based ceragenin that has been used to address common central line-associated bloodstream infections and to protect endotracheal tubes from microbial colonization, demonstrating potent activity against a variety of pathogens [25,26,27]. CSA-131 has also recently been designated as a Qualified Infectious Disease Product for use in the treatment of cystic fibrosis [28]. CSA-44 is a representative ester-based ceragenin that is relatively cheap to produce and has also been screened against a broad range of pathogens and studied in multiple medical applications [27,29,30,31]. Both compounds have been produced at the multi-kilogram scale and are stable at physiological temperatures.

The development of a ceragenin-containing adhesive began with studies of how ceragenins CSA-131 and CSA-44 could be incorporated directly into commercially available, medical-grade adhesives. Polyacrylate adhesive Aroset 700 Gentle (Ashland, Wilmington, DE, USA) was selected for development because it readily formed homogeneous solutions with concentrated solutions of these ceragenins in ethanol. After removal of the ethanol, the resulting adhesive remained stable with desirable adhesive properties, even with relatively large percentages of ceragenins. To incorporate the ceragenin into the acrylate adhesive, the ceragenin was first dissolved in ethanol (e.g., 35% (*w*/*v*)). The ethanol solution containing either CSA-44 or CSA-131 was then mixed with the adhesive in a two-to-one ratio to produce a homogenous solution. This solution was spread to a thickness of 200 µm on capped “release paper”. The adhesive coating was allowed to thoroughly dry before a substrate was added on top of the adhesive layer with gentle pressure. The used substrate was a polyamide/elastane pad acquired from Mederma. The combination of the adhesive on the substrate yielded coated materials POLYA44 and POLYA131 (with POLYAC as the pad material containing the adhesive without ceragenin).

In addition to applied adhesives, we explored other potential uses for ceragenins in adhesive applications. One attractive application was found to be the use of a ceragenin in adhesive silicone scar tape, which is used on postoperative wounds to reduce infection, as well as to reduce the production of scars. Colonization of scar tape has previously been reported as a risk factor in HAIs with respect to patient outcomes [32]. Silicone scar tapes are typically made with one face of the tape comprising capped silicone and the opposite side presenting uncapped silicone. Uncapped silicone has adhesive properties, and no additional adhesive is required to secure the tape to tissue. Thus, a coating was not required for ceragenin incorporation. Rather, the ceragenin was infused directly into the silicone. Silicone swells in the presence of organic alcohols, and exposure of scar tape to CSA-131 in isopropanol resulted in the infusion of the ceragenin into the silicone tape. To accelerate this process, we found that use of intense sonication resulted in greater amounts of the ceragenin in the silicone material. After exposure to CSA-131 in isopropanol and horn sonication, samples were allowed to dry, which allowed the silicone to resume its original shape, trapping the ceragenin within the silicone. Samples were then rinsed with isopropanol to remove residual CSA-131 on the surface. Optimization of this method yielded a material termed SCART131, whereas the untreated scar tape was designated as the scar tape control (SCARTC).

### 2.2. Antimicrobial Properties of Adhesive Materials

**Figure 1:**
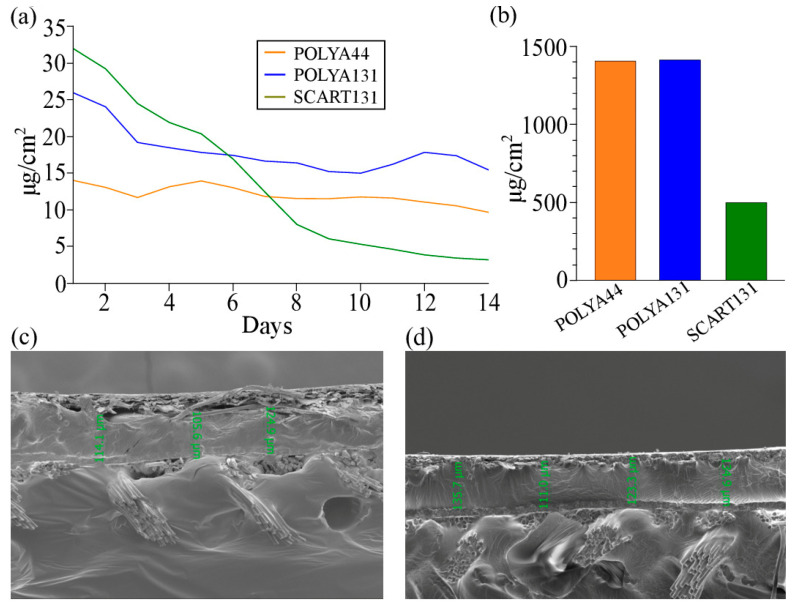
Characterization of ceragenin-containing adhesives. Polyacrylate adhesives containing CSA-44 (POLYA44), polyacrylate adhesive containing CSA-131 (POLYA131), and scar tape containing CSA-131 (SCART131). (**a**) 3-day rolling average of daily elution of CSA-131 and CSA-44 from medical adhesives. (**b**) Total extraction of CSA-44 and CSA-131 from medical adhesives. (**c**,**d**) SEM imaging of POLYA131 (**c**) and POLYA44 (**d**) with measurements of adhesive thickness. Release paper is visible above the adhesive, and the substrate pad is visible below.

To verify that the ceragenin reservoirs would be accessible and released in a controlled manner, we ran elution assays in phosphate-buffered saline (PBS). Samples were immersed in PBS and incubated at 37 °C for 24 h. Samples were moved to fresh PBS, and the process repeated. The amounts of ceragenins that eluted daily are plotted in Figure 1a as the amount released per square centimeter. SCART131 and POLYA131 yielded the highest elution on the first day, which may be important in eliminating existing microbes at sites of application. Notably, the ceragenins continued to elute for more than 14 days, which was expected to provide a defense against colonization by new microbes. POLYA44 showed a near zero-order release over the 14-day evaluation.

To measure the total amounts of ceragenin in the adhesives and scar tape, sections of adhesive-coated materials or scar tape were soaked in a mixture of isopropanol and dilute aqueous acid to allow for the complete release of CSA-44 or CSA-131 into the solution. Both POLYA44 and POLYA131 contained approximately 1400 µg/cm^2^ of the ceragenins (Figure 1b). SCART131 had a total CSA-131 extraction of approximately 500 µg/cm^2^ (Figure 1b). This lower amount is attributed to the fact that the ceragenin was infused into silicone rather than being incorporated into a coating. Scar tape is typically used for relatively short timespans [6], so this lower amount was expected to be sufficient for most applications.

We investigated the polyacrylate adhesives for uniformity of the coating and to confirm coating thickness through SEM images of POLYA44 and POLYA131 (Figure 1c,d). To obtain the desired images, coated materials were frozen, then broken sharply. Although the sample of POLYA131 displayed some artifacts of the cracking process used to prepare the sample, such as some delamination and loss of integrity in the release paper, there were no visible salt crystals or bubbles in either sample. The coatings appeared relatively uniform, with a thickness of approximately 120 µm.

The primary objective was to generate adhesive materials with broad-spectrum antimicrobial properties, including activity against representative fungi and Gram-positive and Gram-negative bacteria. We chose MRSA (ATCC BAA-41) as a representative Gram-positive organism, as it is a common pathogen in nosocomial infections. We chose multi-drug-resistant *P. aeruginose* (ATCC 47085) as the Gram-negative bacterium and *C. albicans* (ATCC 90028) as a representative fungal pathogen commonly found in HAIs.

**Figure 2:**
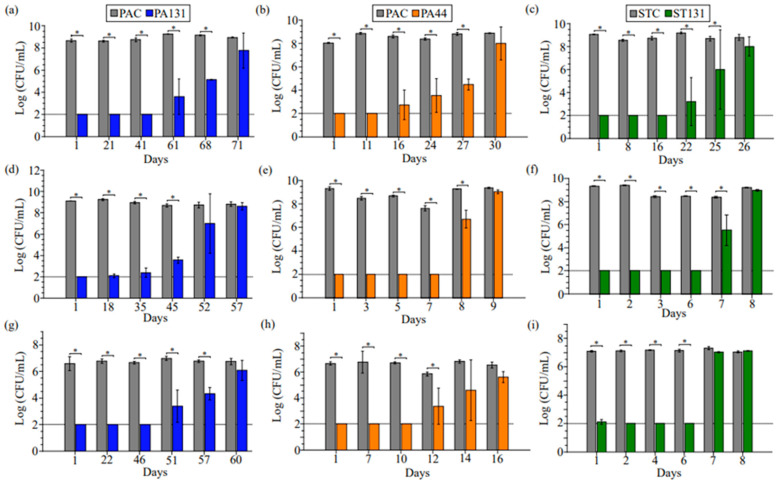
Antimicrobial efficacy of adhesives and scar tape infused with CSA-44 or CSA-131. Daily growth of (**a**–**c**) MRSA, (**d**–**f**) *P. aeruginosa*, and (**g**–**i**) *C. albicans* in the presence of POLYA44, POLYA131, or SCART131. The unaltered adhesive on the same substrate (POLYAC) or uninfused scar tape (SCARTC) were used as controls. Tests were run in triplicate, with error bars indicating the standard deviation. * *p* < 0.05 according to Student’s *t* test. Limit of detection (2 logs CFU/mL) indicated by a horizontal line.

We challenged adhesives POLYA131, POLYA44, and scar tape SCART131 against all three pathogens to determine how long the eluted antimicrobials would control fungal and bacterial growth. Samples were challenged daily with the indicated organisms, with daily exchange of growth medium. Both POLYA adhesives and the adhesive silicone performed best against MRSA, with POLYA131, POLYA44, and SCART131 showing significant reductions in bacterial growth relative to controls for 68, 27, and 25 days, respectively (Figure 2a–c). Against PA, significant bacterial growth reduction was observed for 45, 8, and 7 days, respectively. Significant reductions in fungal growth were observed for 57, 12, and 6 days, respectively, with *C. albicans*. The loss of activity against *P. aeruginosa* and *C. albicans* with POLYA44 and SCART131 on days 6–8 is consistent with lower amounts of ceragenins CSA-44 and CSA-131 eluting on these days (Figure 1a). The correlation of the amounts of ceragenin released from POLYA44 and SCART131 with antimicrobial activity provides information about the absolute amounts of ceragenins required to eliminate the microbial inocula. Notably, the level of CSA-131 released from POLYA131 remained higher than the level of ceragenin released from the other materials, and this material demonstrated the longest-lasting antimicrobial activity. In addition, CSA-131 is active at lower concentrations than CSA-44 against the organisms used in the evaluation of the adhesive materials [27,29,30], and the differences in duration of activity may be influenced by the native activity of ceragenin. Overall, a reduction in growth for all tested strains for at least six days is a promising benchmark, and a reduction in MRSA for three weeks is particularly notable, given its prevalence in HAIs.

### 2.3. Surface Sterilization

Antimicrobial assays in nutrient media are a common means of evaluating the antimicrobial efficacy of substances and devices; however, most practical applications of adhesives take place on surfaces rather than in aqueous media. To test how the adhesives performed in disinfecting a surface, we performed antimicrobial assays on agar. Inocula for each pathogen were spread across fungal or bacterial culture plates to generate a microbial lawn. Adhesive materials were placed on top of the agar, and the plates were incubated overnight. The agar surface underneath the adhesive materials was collected for quantification of surviving pathogens.

**Figure 3:**
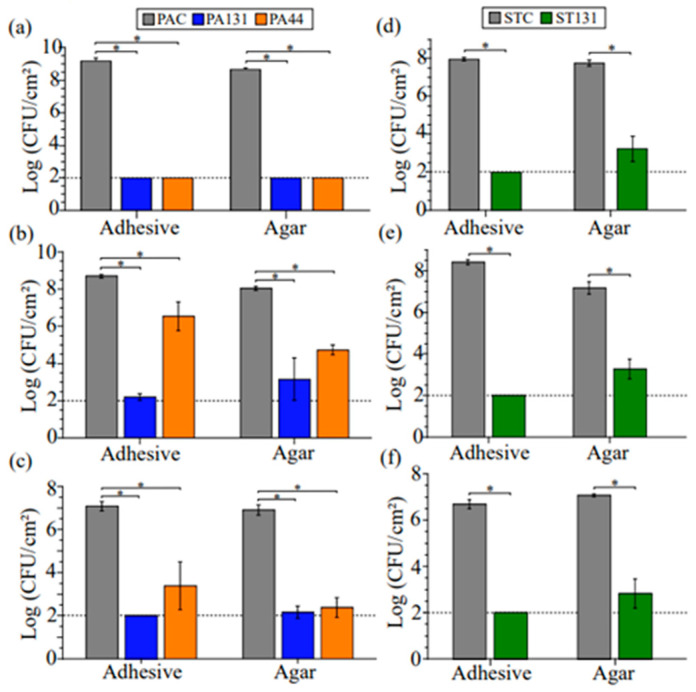
Surface-disinfecting properties of adhesives. Quantification of (**a**,**d**) MRSA, (**b**,**e**) *P. aeruginosa*, and (**c**,**f**) *C. albicans* on adhesive materials and on agar beneath adhesive materials applied to agar lawns of microorganisms. (**a**–**c**) Performance of control (POLYAC) with POLYA44 or POLYA131. (**d**–**f**) Scar tape control (SCARTC) compared with scar tape impregnated with CSA-131 (SCART131). Tests were run in triplicate, with error bars indicating the standard deviation. * *p* < 0.05 according to Student’s *t* test. Limit of detection (2 logs CFU/cm^2^) indicated by a horizontal line.

All adhesive materials significantly reduced the growth of all pathogens (Figure 3). Consistent with results in aqueous media (Figure 2), the POLYA44 and POLYA131 adhesive materials performed best against MRSA, reducing surviving cells to the limit of detection (Figure 3a). With *P. aeruginosa* and *C. albicans*, growth was reduced by more than three logs by both POLYA44 and POLYA131 relative to controls (Figure 3b,c). Similarly, SCART131 yielded a more than three-log reduction in all pathogens relative to controls (Figure 3d–f). Due to the possibility that the adhesive could remove surviving cells, we also evaluated the removed adhesives for the presence of bacteria or fungi. All adhesives remained free of MRSA contamination (Figure 3a,d) and reduced *P. aeruginosa* and *C. albicans* contamination by more than four logs (Figure 3b,c,e,f). These results suggest that ceragenin-containing adhesive materials may prevent the cross-contamination observed in previous studies of medical adhesives [8,9].

### 2.4. Adhesive Strength Testing

**Figure 4:**
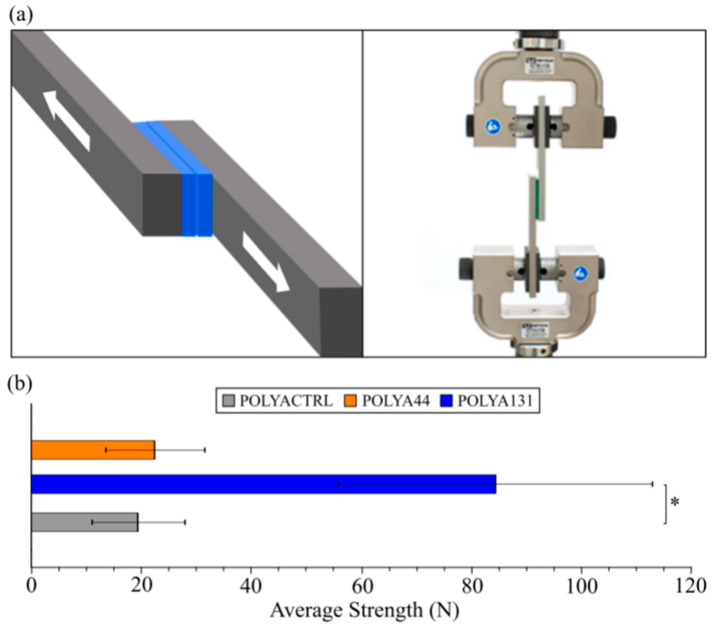
Impact of ceragenins on shear adhesive strength. (**a**) Schematic illustration of the lap shear test. (**b**) Shear strength measurements of polyacrylate adhesive controls (PACs), POLYA44 or POLYA131. Error bars represent the standard deviation. * *p* < 0.05.

An important characteristic of adhesives in a medical setting is the ability to adhere at a strength that will keep a device anchored. The ability to prevent slipping is best measured by the shear strength of the adhesive, and losses in shear strength could be detrimental to a novel adhesive formulation [33,34]. We aimed to determine if the presence of CSA-44 and CSA-131 affected the adhesive strength by performing a lap shear test (Figure 4a). In this assay, the force required to cause failure of the adhesive was measured. With the parent adhesive (PAC), the required force was ca. 20 N (Figure 4b). A comparable force was required for POLYA44 (ca. 22.5 N). The presence of CSA-131 in POLYA131 increased the strength of the adhesive, requiring 84.5 N. Thus, there was no significant difference between the adhesive without any antimicrobial agent and that with CSA-44. Interestingly, adding CSA-131 significantly increased the adhesive strength, demonstrating a potential improvement in preventing adhesive movement. The values of force were not intended to relate to actual medical conditions. Assays were not performed under clinical conditions.

### 2.5. Dermal Irritation Measurements

The primary application of adhesive materials is expected to be on skin, and to ensure that the adhesives containing ceragenins did not cause irritation, the biocompatibilities of adhesive materials POLYA44 and POLYA131 were measured. Application locations on New Zealand white rabbits were shaved, and POLYA44 and POLYA131 patches were applied. The standard protocol for skin irritation evaluation (protocol ANSI/AAMI/ISO 10993-23-2021) calls for a test material residence time of 4 h. Because the use of ceragenin-adhesive materials is expected to last for longer periods, materials were left in place for 24 h. At 24, 48, and 72 h, sites of POLYA44 and POLYA131 application were evaluated for erythema and edema caused by the materials using scores of 0–4 (no erythema/edema (0) to severe erythema/edema (4)). Both adhesive materials (POLYA44 and POLYA131) were characterized as non-irritating, with scores of 0. The amount of CSA-131 in scar tape was less than half that found in POLYA131, and initial release rates of CSA-131 from POLYA131 and SCART131 were comparable; therefore, SCART131 is also expected to be non-irritating.

## 3. Discussion

Medical adhesives and silicon-based scar tape are vital to the medical community as a means of protecting compromised skin from the environment and to secure medical devices extending through the skin; however, they can serve as nidi for nosocomial infections. Post-operative infections from procedures such as cesarean sections, as well as IVs and unsterile adhesives, are responsible for many deaths [6,7]. Owing to the combination of *S. aureus* and other bacterial infections, skin-associated HAIs cost the healthcare industry almost USD 3 billion and thousands of lives annually [1]. These occur despite local sterilization at incision sites and at sites at which medical devices pass through the skin. High rates of infection persist even with preoperative antibiotic prophylaxis [5,35,36], and bacterial infection is a common wound complication that can significantly delay healing. Many of these infections may be preventable by providing adhesive coverings that augment and/or replace the naturally occurring innate immune functions of the skin. As noted, the innate immune function produced within the skin offers broad-spectrum antimicrobial protection. The breadth of the spectrum of this activity is essential because Gram-positive and Gram-negative bacteria and fungi are ubiquitous in the environment, and their growth must be controlled to prevent infiltration of these microorganisms into underlying tissues.

There have been other attempts to incorporate broad-spectrum antimicrobials into wound dressing; for example, silver nanoparticles have been used with limited results [37]. The incorporation of silver nanoparticles into chitin dressings reduced the growth of SA and PA in a broth challenge, but bacteria persisted after 24 h [38]. Silver nanoparticles were formulated in cellulose as a delivery mechanism, but the best formulation only achieved a two-log reduction in *S. aureus* [39].

Ceragenins are uniquely suited to provide broad-spectrum, innate, immune-like protection for damaged skin. They display high levels of activity against Gram-positive and Gram-negative bacteria and fungi, and they can be readily incorporated into adhesive materials, including polyacrylate adhesives and adherent silicone. By controlling the amounts of ceragenins that are incorporated into adhesive materials, they elute over extended periods, providing protection that can persist for weeks. Comparison of the amounts of CSA-44 and CSA-131 eluting from these materials with previously reported minimum inhibitory concentrations (MICs) for ceragenins against other bacteria and fungi suggests that these materials may be effective in controlling the growth of other pathogens for similar periods of time [27,28,29,30]. The microbial selectivities of ceragenins have been the subject of multiple studies consistent with the greater susceptibility of Gram-positive bacteria reported here [40]. Ceragenins typically display lower MICs against Gram-positive bacteria due to the direct access that the compounds have to the cytoplasmic membrane, while activity against Gram-negative bacteria requires the compounds to traverse the outer membrane.

Additionally, the presence of ceragenins does not decrease the adhesive properties of the materials. Research focusing on the physical properties of ceragenin-containing adhesives is ongoing and will provide additional insights into the molecular interactions of adhesive additives [41]. The amounts of ceragenins eluting from these acrylate adhesives is non-irritating, and previous research has shown that ceragenins promote cell migration into wound beds in the same manner as endogenous AMPs in human skin [27]. This stimulation of cell migration combined with the antimicrobial activities of ceragenins is expected to reduce hypertrophic scar formation and accelerate wound closure.

## 4. Materials and Methods

### 4.1. Preparation of Antimicrobial Polyacrylate Adhesives

Aroset^TM^ Gentle 700 pressure-sensitive adhesive was purchased from Ashland (Wilmington, DE, USA), comprising 45% solids (*w*/*w*). Solutions of the HCl salts of CSA-44 and CSA-131 were prepared in ethanol at 35% (*w*/*v*). The prepared ceragenin solutions were added to the Aroset Gentle 700 in a 1:2 ratio (*v*/*w*). The ceragenin solutions and adhesive were mixed for 1 h until visibly homogenous. The mixtures were poured onto silicone release paper and spread using a Gardco^®^ Universal Blade Film Applicator (Gardco, Columbia, GA, USA) set to 200 µm to create a uniform layer. All adhesives were dried for 72 h within a SafeAire fume hood (Fisher Hamilton Scientific, Two Rivers, WI, USA) at room temperature to allow volatile substances to evaporate, leaving a clear, tacky layer on the release paper onto which substrates could be placed. The absence of ethanol after 72 h was confirmed by Raman spectroscopy. A polyester cloth (VWR International, Radnor, PA, USA) was used as the substrate pad for antimicrobial broth testing, and a polyamide/elastane blend (Mederma, Raleigh, NC, USA) was used as a substrate for imaging and surface disinfection assays. The cloth substrates were sterilized by immersion in isopropanol for 60 s, followed by immersion in hexanes for 30 s. The cleansed substrate was allowed to dry for 24 h at room temperature before being placed on top of the adhesive with light pressure. Sections for extraction, elution, and antimicrobial broth evaluation were formed using a 10 mm square metal die.

### 4.2. Preparation of Antimicrobial Scar Tape

To prepare the ceragenin-containing scar tape, sections of Mederma Scar Sheets (Mederma, Raleigh, NC, USA) were punched in 1 cm squares using a template metal die. After removing the release paper covering the adhesive face, squares were immersed in a 1% CSA-131 (*w*/*v*) isopropanol solution. The solution and scar tape were sonicated with a Branson Digital Sonifier SFX 550 horn sonicator (St. Louis, MO, USA) for 15 min at 275 watts. After sonication, scar-tape samples were removed from the solution and air-dried for 12 h with the uncapped side facing up. Each scar-tape square was rinsed in 10 mL of isopropanol for 2 min to remove surface ceragenin. The scar tape was then dried again for 12 h, and release paper was reapplied to the adhesive surface.

### 4.3. Total Extraction of Ceragenins

The total amounts of CSA-131 and CSA-44 loaded into adhesive materials were determined by immersing 10 mm adhesive squares, with release paper removed, into a solution of 20% 1 N aqueous HCL in isopropanol at 60 °C. The extraction solution was collected and replaced regularly, with the first three fractions collected every 8 h, followed by four fractions collected after 24 h intervals at 37 °C. After each fraction was collected, the concentrations of CSA-131 and CSA-44 were determined on an Agilent (Santa Clara, CA, USA) 6530 QTOF with connected 1260 HPLC using mass-labeled internal standards (CSA-131D_25_ and CSA-44D_2_). These internal standards were prepared in the same manner as their non-labeled counterparts, except that deuterated starting materials were employed. The calculated concentrations of the seven fractions were summed for each sample set. Sample extractions were run in triplicate, with error bars representing standard deviations among extraction totals. To validate the methods used to quantify ceragenin concentrations, internal mass labeled standards were calibrated using freshly prepared solutions of the ceragenin analytes generated from pure (>97%) ceragenins.

### 4.4. Quantification of Ceragenin Elution

Square sections (10 mm) of adhesive materials containing CSA-131 and CSA-44 were prepared as described above. Release paper was removed, and samples were placed in 5 mL conical tubes and immersed in PBS (1 mL). The conical tubes were placed in an incubator at 37 °C for 24 h. After the incubation period, the PBS solution was removed, and equal parts of the collected solution and a deuterated internal standard were mixed. The mixture was evaluated on an Agilent 6530 QTOF with connected 1260 HPLC to determine the concentration of ceragenin eluted over the 24 h period of incubation. Deuterated standards (CSA-131D_25_ and CSA-44D_2_) were prepared and validated as described above and added to samples to serve as internal standards for quantification. At 24 h intervals, sections were rinsed once with PBS and submerged in fresh PBS. The process was repeated daily for 2 weeks.

### 4.5. Scanning Electron Microscopy

Adhesive material strips were immersed in liquid nitrogen to allow for fracturing. After 15 min, strips were removed and snapped by bending. The samples were mounted on SEM pin stubs and sputter-coated with approximately 20 nm of a gold–palladium alloy using a Quorum Q 150T ES (Electron Microscopy Sciences, Hatfield, PA, USA). The SEM stub was loaded at a 45° chamfer in the mounting stage. The stage was further tilted by an additional 45° to allow for normal imaging of the snapped cross-section of the adhesive strip. Images and measurements of the adhesive cross-sections were obtained using an Apreo C microscope (Thermo Fisher Scientific, Waltham, MA, USA).

### 4.6. Microbial Cultures

Bacterial and fungal cultures were prepared from fresh colonies placed in media and incubated overnight at 37 °C. *P. aeruginosa* (PA01, ATCC 47085) and MRSA (ATCC BAA-41) were cultured in trypticase soy broth (TSB). *C. albicans* (ATCC 90028) was cultured in Emmons-modified Sabouraud dextrose broth (EMSDB). The cultures were centrifuged and rinsed three times in phosphate-buffered saline (PBS) and resuspended in PBS. Optical density (OD) readings were performed at 600 nm on a Genesys 30 spectrophotometer (Thermo Fisher Scientific, Waltham, MA, USA) to determine cell density. Cell suspensions were diluted to form inocula of 10^3^ CFU/mL in 10% TSB in PBS and 10^3^ CFU/mL in 10% EMSDB in PBS for bacteria and fungi, respectively.

### 4.7. Evaluation of Antimicrobial Properties of Adhesives

Adhesive materials were prepared as described above, and a 10 mm square metal cutting die (Walfront, Lewes, ED, USA) was used to generate identical sections for microbial challenge. Release paper was removed, and adhesive material sections were placed individually in wells of a 12-well plate, immersed in 1 mL of inoculum, and incubated for 24 h. Microbial growth was quantified by removing 20 µL aliquots and adding them to Dey–Engley neutralizing broth (Sigma-Aldrich, St. Louis, MO, USA). The resulting suspensions were serially diluted in a 96-well plate, and 100 µL aliquots of the resulting suspensions were spread on nutrient agar. Bacterial plates were incubated for 24 h, and fungal plates were incubated for 48 h before colonies were counted. After sampling, the remaining growth medium was removed from the adhesive sections. The sections were washed three times with PBS, transferred to new 12-well plates, immersed in nutrient media, and inoculated with fresh bacterial or fungal culture. Samples were incubated for an additional 24 h. This process was repeated every 24 h. The study was run in triplicate, and growth was measured daily until statistical significance of microbial reduction, relative to controls, was lost as determined by Student’s *t* test (*p* < 0.05). The detection limit for these studies is based upon the volume of solution plated on agar. The lowest number of organisms that can be accurately quantified is 100 CFU/mL. Thus, results reported as two logs of bacteria or fungi can range from 0 to 100 organisms per milliliter. *t* tests were performed using Microsoft Excel (version 16.90).

### 4.8. Surface Sterilization

Antimicrobial efficacy of surfaces, including adhesive surfaces, can be evaluated by direct application of bacterial or fungal cultures (for example, see Ref. [42]). However, the clinical use of adhesive materials involves the placement of the materials onto surfaces containing established microbial colonies. Consequently, we chose to evaluate the ceragenin-containing adhesives by placing them on an established lawn of microorganisms. Circular samples of each test adhesive material with a radius of 10 mm were generated using a metal cutting die. A lawn of the indicated pathogen was generated by streaking agar plates with a cotton swab dipped in a 5 × 10^7^ CFU/mL suspension. Release paper was removed from the adhesive samples, which were then placed onto the prepared plates. Plates were allowed to incubate for 24 h. After the incubation period, the adhesive materials were removed and placed into neutralizing broth, sonicated for 10 min, and vortexed for 1 min. The resulting suspension was serially diluted, spread on agar plates, and incubated. The agar under the adhesive was also sampled, with a 10 mm square section being removed; placed in neutralizing broth; and sonicated for 10 min to release surviving cells. The resulting suspension was serially diluted, spread on agar plates, and incubated. Colonies were quantified after 24 h for bacteria and after 48 h for fungi. *t* tests were performed using Microsoft Excel.

### 4.9. Single-Lap Shear Test

A single-lap shear test is a common method for evaluating adhesive strength [33,43]. In this test, a single layer of adhesive is sandwiched between two metal or polymer substrates, and a shear load is applied parallel to the plane of the substrates (Figure 4a). The test provides a load–displacement curve to determine the shear strength of the adhesive bond following the ASTM D4896-01 standard [43]. The adhesive strength without any additive was evaluated against the adhesive strength with the addition of the two different ceragenins. A 50 mm × 25.7 mm × 0.08 mm layer of adhesive was placed between two 73 mm × 25.7 mm × 6.5 mm high-density polyethylene (HDPE) substrates and pressed for 2 h under 1.5 lb of weight. The HDPE substrates were then separated at a rate of 5 mm/min using a 3345 series Instron tensile strength tester to measure the load and extension. The maximum load values for the samples were averaged to obtain a representative strength of adhesion for each adhesive formulation.

### 4.10. Dermal Irritation Study in Rabbits

The skin irritation potential of adhesive patches containing either CSA-44 (POLYA44) or CSA-131 (POLYA131) was evaluated following protocol ANSI/AAMI/ISO 10993-23-2021, with the following modifications: adhesive patches remained in place for 24 h (rather than 4 h in the protocol), and patch sizes were 2 × 2 cm (rather than 2.5 × 2.5 cm in the protocol). The study was performed at Geneva Laboratories (Elkhorn, WI, USA) with New Zealand white rabbits (*Oryctolagus cuniculus*) under IACUC approval from a standing committee at Geneva Laboratories (CL1024 revision Q). Briefly, the backs of three animals were clipped free of hair to expose 15 × 15 cm of skin. Two control patches—two containing CSA-44 and two containing CSA-131—were applied to each animal. To ensure that patches remained in place, they were covered with a self-adhering wrap and a cloth stocking. After 24 h, coverings and patches were removed, and the covered skin was evaluated for erythema and edema using scores of 0–4, from no erythema/edema (0) to severe erythema/edema (4). Evaluation was performed by a trained pathologist at 24, 48, and 72 h. Skin under the patches, for both control and test samples, was scored as 0 at all time points. To validate the protocol, a positive validation test was performed at Geneva Laboratories every six months using sodium dodecyl sulfate in petroleum jelly, which scores as a “moderate” irritant.

## 5. Conclusions

Herein, we have demonstrated two methods of incorporating ceragenins into pressure-sensitive adhesives and silicone scar tape. By either loading the compounds into precursor solutions or impregnating completed devices through sonication, stable and effective combination adhesives can be created. These adhesives exhibit steady elution of the active antimicrobial agents for multiple weeks and reduce the growth of bacterial and fungal pathogens on adhered surfaces by more than three logs. This efficacy, combined with a lack of dermal irritation while maintaining or improving lap shear strength, make these adhesives highly suited for multiple applications.

## Data Availability

The data presented in this study are available in this article. Further original data can be obtained from the corresponding author upon reasonable request.
